# Post-carotid stenting reperfusion injury with blood–brain barrier disruption on gadolinium-enhanced FLAIR MRI

**DOI:** 10.1186/s12883-014-0178-z

**Published:** 2014-09-10

**Authors:** Hyun-Ji Cho, Young Jin Kim, Joon Hwa Lee, Jin Woo Choi, Won-Jin Moon, Hong Gee Roh, Young Il Chun, Hahn Young Kim

**Affiliations:** Department of Neurology, Konkuk University School of Medicine, 120-1 Neungdong-ro, Gwangjin-gu, Seoul, 143-729 Republic of Korea; Department of Neurology, The Catholic University of Korea, St. Mary’s Hospital, Incheon, Republic of Korea; Department of Radiology, Konkuk University School of Medicine, Seoul, Republic of Korea; Department of Neurosurgery, Research Institute of Medical Science, Konkuk University School of Medicine, Seoul, Republic of Korea

**Keywords:** Reperfusion injury, Carotid stenting, Blood–brain barrier, MRI

## Abstract

**Background:**

Following carotid revascularization, an abrupt increase in cerebral blood flow may disrupt the blood–brain barrier, resulting in reperfusion injury. This damage to the blood–brain barrier may be reflected by subarachnoid enhancement on FLAIR MRI after gadolinium injection.

**Case presentation:**

The authors present two cases of post-carotid stenting reperfusion injury that showed hyperintensity in the subarachnoid spaces on FLAIR MRI after gadolinium injection.

**Conclusion:**

These MRI findings may represent a marker for reperfusion injury after carotid revascularization.

## Background

The mechanism of hyperperfusion syndrome (HPS) occurring after carotid endarterectomy or carotid artery stenting (CAS) may involve disruption of the blood–brain barrier (BBB) induced by abrupt increases in cerebral blood flow [1]. Patients with HPS present a variety of clinical manifestations, including headache, visual disturbance, confusion and other hemispheric symptoms [1,2]. Recently, extravasation and stagnation of intravenous gadolinium (Gd) in the subarachnoid space has been suggested to be an imaging marker for early BBB disruption in ischemic stroke. This has been denominated as a “hyperintense acute reperfusion marker (HARM)” [3]. Herein, two patients with post-carotid stenting HPS and HARM are presented.

## Case presentation

### Serial T2 FLAIR MRI protocol

To detect Gd extravasation and stagnation in the subarachnoid space, serial fluid-attenuated inversion-recovery (FLAIR) MRIs were performed using a protocol similar to that reported previously [4,5]. FLAIR MRIs were performed thrice. First, pre-stenting FLAIR MRI was performed before CAS. Gd was then injected 6–8 hours after CAS to perform perfusion-weighted MRI (PWI). In patient 1, an additional FLAIR MRI was performed immediately after Gd injection. Second, post-stenting FLAIR MRI was performed 24–30 hours after CAS (i.e. 18–24 hours after Gd injection). Third, FLAIR MRIs were performed 4–5 days after CAS.

### Patient 1

**Figure 1:**
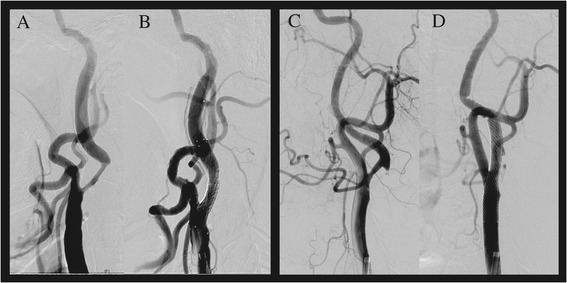
**Pre and post carotid stenting.** Conventional cerebral angiography showed > 70% stenosis of the left proximal carotid artery **(** **A** **and** **C** **)**. Carotid artery stenting was successfully performed **(B and D)**. (**A** and **B** in patient 1; **C** and **D** in patient 2).

**Figure 2:**
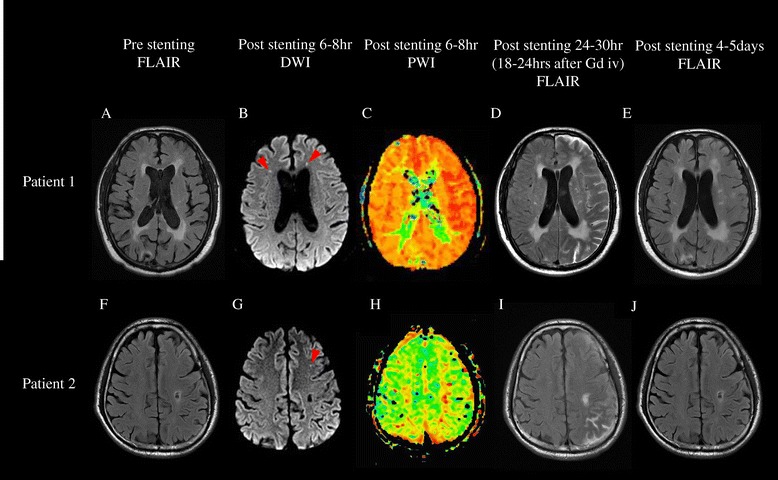
**Serial follow-up FLAIR MRI.** Chronic ischemic white matter changes were observed in pre-stenting FLAIR MRI **(A and F)**. Post-stenting FLAIR MRI performed 18–24 hours after intravenous Gd showed hyperintensities in the subarachnoid space **(D and I)**, which resolved in 4–5 days **(E and J)**. Post-stenting DWI showed a few small subcortical lesions (arrowheads in **B** and **G**). Slightly increased perfusion on the time-to-peak map in the left hemisphere was observed in patient 1 **(C)**.

**Figure 3:**
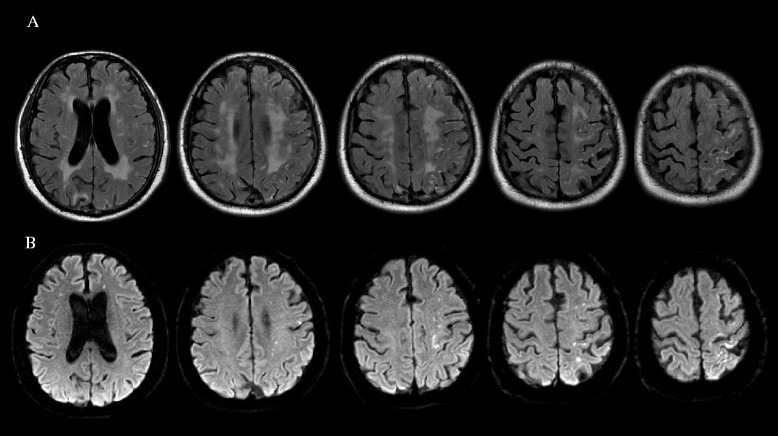
**Immediate FLAIR MRI after Gd injection showed diffuse leptomeningeal enhancements along the cerebral cortex of the left hemisphere (A) accompanied by signal changes in DWI (B) in patient 1.**

A 67-year-old man with hypertension and diabetes mellitus was admitted for transient aphasia. Diffusion-weighted MRI (DWI) performed 5 hours after the onset of symptoms did not reveal the presence of acute infarction in the left hemisphere. MR angiography and conventional cerebral angiography revealed > 70% stenosis of the left proximal internal carotid artery (ICA) (Figure 1A). CAS of the left proximal ICA was successfully performed with a distal protection device at 7 days after the onset of symptoms (Figure 1B). Three hours later, he was disoriented, agitated, and experienced sensory aphasia and drift of his right arm. Systolic blood pressure was maintained between 129 and 163 mmHg, and diastolic blood pressure was maintained between 90 and 107 mmHg, values that were slightly higher than the pre-stenting blood pressure. DWI performed 6 hours post-stenting showed several small subcortical infarctions in the frontal subcortex, probably associated with the stenting procedure (Figure 2B). PWI performed 6 hours post-stenting showed mildly increased perfusion on the time-to-peak map in the left hemisphere (Figure 2C). Immediate FLAIR MRI after Gd injection showed diffuse leptomeningeal enhancement along the cerebral cortex in the hemisphere with hyperperfusion (Figure 3A) that was accompanied by signal changes on DWI (Figure 3B). Follow-up FLAIR MRI performed 18 hours after Gd injection showed subarachnoid hyperintensities in the left cerebral hemisphere (Figure 2D). The patient recovered in 5 days and experienced only mild dysarthria. Subarachnoid hyperintensities were completely resolved on follow-up FLAIR MRI (Figure 2E). Increased perfusion in the left hemisphere was nearly normalized on the follow-up PWI, which was performed 24 hours after stenting.

### Patient 2

A 66-year-old man with hypertension and diabetes mellitus was admitted for left proximal internal carotid arterial stenosis. He had presented 1 week earlier with mild dysarthria that had completely resolved. DWI showed several small acute lesions in the left hemispheric borderzone; therefore, the carotid stenosis was symptomatic. MR angiography and conventional cerebral angiography revealed 70% stenosis at the bifurcation of the left ICA (Figure 1C). CAS of the left proximal ICA was successfully performed with a distal protection device (Figure 1D). Blood pressure was strictly controlled during and after the procedure. Five hours later, he was disoriented and agitated with aphasia. DWI performed 8 hours after stenting showed several small cortical infarctions in the left frontal subcortex (Figure 2G). PWI performed 8 hours after stenting showed no perfusion abnormalities (Figure 2H). One day later, his aphasia worsened. Follow-up FLAIR MRI performed 24 hours after Gd injection showed subarachnoid hyperintensities in the left cerebral hemisphere (Figure 2I). No new lesions were revealed on follow-up DWI. The patient completely recovered in 4 days. Subarachnoid hyperintensities appeared to be completely resolved on follow-up FLAIR MRI (Figure 2J).

## Discussion

An abrupt increase in cerebral blood flow following revascularization has been identified as the direct physiological cause of HPS [1]. Impaired autoregulation of cerebral blood flow and subsequent disruption of the BBB are possible conditions associated with HPS [1,2]. Leptomeningeal enhancement on Gd-enhanced FLAIR MRI has been observed in patients with meningitis, subarachnoid hemorrhage (SAH), leptomeningeal carcinomatosis or renal dysfunction [6,7]. First, the patients described herein did not show any clinical symptoms of SAH or meningitis such as severe headache or neck stiffness, and had no evidence of intracranial aneurysms or infection. Sulcal FLAIR hyperintensities, which were not observed in either patient before stenting (Figure 2A and F), appeared after Gd injection and rapidly disappeared in 4–5 days (Figure 2E and J). In cases of SAH or meningitis, sulcal FLAIR hyperintensities may be present before stenting and remain for longer periods of time.

Hyperintensities on FLAIR MRI after Gd enhancement have been suggested to be a marker of reperfusion injury after thrombolysis [3,8,9]. Serial pre- and post-stenting Gd-enhanced FLAIR MRI studies in patients with CAS have revealed leptomeningeal enhancement after stenting [10,11]. Wilkinson et al. reported asymptomatic leptomeningeal enhancements that were the consequence of hemodynamic changes after CAS [11]. Because that study only involved patients with symptomatic carotid stenosis, the underlying disruption of the BBB by previous ischemic injury may have resulted in leakage of the Gd injected. The authors recommended further studies using DWI to clarify these findings [11].

The patients described herein showed clinical symptoms of HPS. Post-stenting DWI showed only a few small ischemic lesions that were probably associated with the stenting procedure; however, these limited lesions do not fully explain the extent of the patients’ hemispheric symptoms. Post-stenting cerebral blood flow measurements by PWI showed mild hyperperfusion on the time-to-peak map in patient 1. The FLAIR MRI performed immediately after Gd injection showed leptomeningeal enhancements along the cerebral cortex; this “on the spot” image may reflect Gd extravasation through the disrupted BBB during the hyperperfusion state (Figure 3A). Interestingly, DWI also showed acute high-signal intensities along the cerebral cortex (Figure 3B). Focal disruption of the BBB in patients with acute ischemic stroke may be the cause of HARM as seen by FLAIR MRI. However, HARM in patients with HPS may be due to transient reversible diffuse hemispheric disruption of the BBB. Although the possibility for multiple microembolic infarctions was present, cortical neuronal injury associated with hyperperfusion is a possible explanation for the lesions observed on DWI. However, considering that pre-stenting stenoses in both patients were less than 80% (74.8% in patient 1 and 70.2% in patient 2), alternative explanations, such as no-reflow or luxury perfusion phenomena, should be considered [12,13].

Extravasated Gd appeared on the follow-up MRI as hyperintensities in the subarachnoid space. After 4–5 days, Gd washout was complete, and the clinical symptoms rapidly improved. In the presence of concomitant acute infarcted lesions in which the BBB is already disrupted, the clinical significance of post-stenting HARM may be quite limited; it could be a simple consequence of Gd leakage through the disrupted BBB in the normal perfusion state, but not in the hyperperfusion state. Recently, similar case and research reports have been published suggesting that reperfusion syndrome may be associated with transient neurological deficits after carotid revascularization without classical HPS [4,5]. Several factors including advanced age, underlying leukoaraiosis, and postprocedural high blood pressure have been associated with symptomatic HARM [4]. Similar to the patients in a previous case series study [5], the patients herein also showed reversible neurological deterioration and limited abnormalities on PWI. Findings on PWI in our patients (mild asymmetry on the time-to-peak map in patient 1 and no asymmetry in patient 2) seemed to be very similar to those in that report (symmetric in 2 patients and mild asymmetry on the mean-transit-time map in one patient) [5].

## Conclusions

HARM may be associated with a mild form of reperfusion injury instead of full-blown HPS. Therefore, further studies considering the multiple factors that are potentially related to post-stenting HARM, such as acute or chronic infarction, white matter hyperintensities, microbleeds, and clinical symptoms of HPS, may be needed.

## Consent

Written informed consent was obtained from the patients for publication of these case reports and any accompanying images. Copies of the written consents are available for review by the editor of this journal.
